# What is the relationship between microorganisms in the human body and upper tract urothelial carcinoma?

**DOI:** 10.3389/fimmu.2025.1636782

**Published:** 2025-10-03

**Authors:** Xin Pei, Minghui Yu, Yijia Wang, Shi Zong, Fei Qi, Kaichen Wang

**Affiliations:** ^1^ Department of Urinary Surgery, China-Japan Union Hospital, Jilin University, Changchun, China; ^2^ Bethune First Clinical Medical College, Jilin University, Changchun, China; ^3^ Department of Operating Room,China-Japan Union Hospital, Jilin University, Changchun, China

**Keywords:** upper tract urothelial carcinoma, urinary microbiome, gut-kidney axis, tumor microenvironment, metabolomics

## Abstract

Upper Tract Urothelial Carcinoma (UTUC) is a highly malignant tumor originating from the epithelium of the upper urinary tract with diverse pathogenesis, but currently available diagnostic and therapeutic strategies have some limitations. In recent years, human microbiome-related studies have provided new ideas for the exploration of the pathogenesis and treatment of UTUC. In this paper, we review the research progress of human microbiome related to UTUC. Focusing on the urinary microbiome, the role of the microbiome in the pathogenesis of UTUC is investigated through the mechanisms of chronic inflammation, genotoxic damage, immune microenvironmental imbalance and metabolic reprogramming. The pyelo-ureteric microbiome of healthy populations is dominated by commensal bacteria such as Lactobacillus and Streptococcus, whereas pathogenic bacteria such as Escherichia coli (E. coli) and Enterococcus faecalis are significantly enriched in patients with UTUC, which results in the development of DNA damage, inflammatory response and immunosuppression. In addition, microbiome metabolites (e.g., short-chain fatty acids, tryptophan derivatives) can influence tumor progression by modulating immune checkpoints (e.g., PD-1/PD-L1, B7-H4) and metabolic pathways (e.g., Warburg effect). In diagnostic and therapeutic applications, urinary microbial markers (e.g., E. coli-specific gene clusters) can be combined with circulating tumor DNA (ctDNA) assays to improve diagnostic sensitivity and specificity, and indices of intestinal flora diversity (e.g., Simpson’s index) are significantly correlated with the response rate to chemotherapy and prognostic course. In the future, we need to overcome the challenges of difficult sample acquisition, unknown causal mechanisms, and etiologic heterogeneity interference, and promote multi-omics joint modeling as well as cross-ethnicity and geographic research, and bidirectional regulation mechanisms of the gut-kidney axis in order to develop more accurate UTUC diagnosis and treatment strategies.

## Graphical Abstract

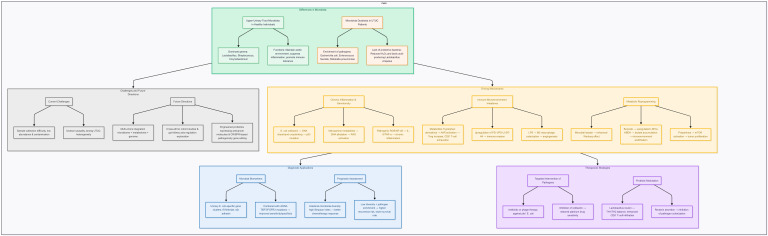

## 1 Introduction

Upper Tract Urothelial Carcinoma (UTUC) is a malignant tumor originating from the epithelium of the renal pelvis and ureter, accounting for 5%-10% of all uroepithelial cancers. The epidemiologic features of UTUC in the Chinese population differ significantly from those in the West, and may be mainly related to exposure to aristolochic acid-containing herbs or foods, with geographic specificity in pathogenesis, clinical features and prognosis (1). Some national data showed that renal pelvic cancer accounted for 31.7% of malignant renal tumors in the same period between 1952 and 1991, and the incidence rate showed a trend of increasing every decade, from 1.9 cases/year (1950s) to 7.8 cases/year (1990s). Although radical nephroureterectomy is the mainstay of treatment, the 3- and 5-year postoperative survival rates are only 75.6% and 60.2%, respectively, and about 16% of patients experience bladder cancer recurrence (2), suggesting that breakthroughs in the existing diagnostic and therapeutic strategies are urgently needed. In recent years, Human Microbiota (HM) research has provided new perspectives on cancer pathogenesis and treatment. Gut microbes influence the efficacy of chemotherapeutic drugs (e.g., platinum) and immunotherapy (e.g., CpG oligonucleotides) through mechanisms such as metabolites (e.g., short-chain fatty acids), immunomodulation (e.g., T-cell differentiation), and modulation of inflammatory responses (e.g., NF-κB pathway) (3). In the field of urologic tumors, the role of the microbiome is gradually gaining attention. Some studies related to bladder cancer have shown that the abundance of Acinetobacter spp. and Bacillus spp. in the urinary flora of patients is significantly elevated, whereas Serratia spp. and Proteus spp. are predominant in healthy populations (4). However, there is still a gap in microbiome studies on pyelo-ureteral cancer, and only a few papers have speculated that urinary flora may be involved in UTUC development through: (i) activation of aristolochic acid metabolism (e.g., intestinal flora-mediated formation of aristolochic acid I → aristolochic acid-lactam-DNA adducts); (ii) chronic inflammation-driven (e.g., persistent activation of the TLR4/MyD88 signaling pathway resulting from flora dysregulation); II immune microenvironment remodeling (e.g., colony metabolites affect PD-1/PD-L1 axis expression) (5). In summary, the aim of this paper is to provide a systematic review of recent research advances in the microbiome of UTUC.

## 2 Differences in the renal pelvic ureteral microbiome

### 2.1 Characteristics of the microbiome in healthy populations

The renal pelvic ureteral microbiome in healthy populations is dominated by commensal bacteria, and typical genera include Lactobacillus (Lactobacillus spp.): which maintains the local acidic environment and inhibits pathogen colonization by secreting lactic acid and hydrogen peroxide (H2O2). Its S-layer proteins (e.g. SlpA/SlpB) can enhance adhesion to epithelial cells and inhibit inflammatory responses by activating the PPAR-γ pathway (6). Streptococcus: some of its strains can maintain ecological homeostasis by competitively inhibiting pathogen adhesion sites (7). Corynebacterium: can promote immune tolerance by modulating the Toll-like receptor (TLR) signaling pathway, e.g., it can inhibit the excessive release of IL-1β and TNF-α (8). These commensal bacteria can maintain local immune homeostasis through metabolites (e.g., short-chain fatty acids) and immunomodulation (e.g., enhanced Treg cell activity) (9). The urinary microbiome has anatomical continuity with the upper urinary tract, but the renal pelvis microbial diversity is significantly lower than that of the bladder, and it possesses certain conditions to form differences with the bladder microbiome. The differences are mainly due to the following factors. Urine flow rate: the relatively fast flow rate of urine in the renal pelvis limits the colonization of microorganisms, while the longer retention time in the bladder is favorable for the proliferation of the flora (10). pH fluctuation: the pH of the urine in the renal pelvis is greatly affected by renal tubular reabsorption, which may inhibit the growth of part of the flora (10). Metabolic environment: metabolites will simultaneously affect the microbiome environment to a certain extent, and higher concentrations of glucose and urea in bladder urine provide growth substrates for commensal bacteria such as Lactobacillus (11).

### 2.2 Characterization of microbiome abnormalities in patients with UTUC

#### 2.2.1 Pathogenic mechanisms of common enriched genera in patients with UTTC

Common enriched genera include Escherichia coli, its subtype can produce Shiga toxin (Stx): toxin-producing strains (e.g., STEC) can cleave the 28S RNA of the ribosome of the host cell through the Stx2 subtype, inhibit protein synthesis and induce cellular growth. The Stx2 subtype inhibits protein synthesis and induces apoptotic mechanisms, leading to the development of renal tubular epithelial damage. Adhesion factor modality: the intimin protein encoded by LEE pathogenicity islands mediates tight adhesion between bacteria and host cells, thereby promoting inflammatory responses (12). Enterococcus faecalis: it produces reactive oxygen species (ROS) via glycerol phosphate oxidase (GPO), which activates the NF-κB pathway and upregulates IL-8 and TNF-α expression, which can induce the onset of oxidative stress. which can further promote biofilm formation: the Esp protein promotes biofilm formation and enhances its antibiotic resistance and persistent infections (13). Klebsiella pneumoniae (K. pneumoniae): releases pro-inflammatory factors, lipopolysaccharide (LPS) activates macrophages via TLR4, secretes IL-6 and IL-1β, and inhibits NLRP3 inflammatory vesicles which in turn leads to chronic inflammation (14). Disruption of the epithelial barrier: its secretion of proteases to degrade tight junction proteins (e.g., occludin), which increases transcellular permeability, can cause some disruption of the epithelial barrier (15).

#### 2.2.2 Deficient flora of the environment

The absence of certain flora of the environment may lead to the development of UTUC. For example, the reduced abundance of the symbiotic bacterium Lactobacillus crispatus showed a correlation with impaired barrier function. Its deficiency leads to a decrease in H2O2 and lactate secretion, allowing local pH to increase and promoting the proliferation of pathogenic bacteria (e.g., E. coli), and its S-layer protein (SlpB) deficiency impairs adhesion and reduces the protective effect on epithelial cells (16–18).

#### 2.2.3 Microbiological differences between utuc and normal tissues

Increase in pro-tumor-associated genera: Acinetobacter, Anaerococcus and Sphingobacterium are significantly more abundant in the urinary microbiota of bladder cancer patients. The genus Streptococcus is significantly enriched in some patients with uroepithelial carcinoma and may be associated with tumorigenesis. Fusobacterium spp. (e.g. Clostridium nucleatum) have been postulated as potential pro-tumor pathogens that may promote cancer progression by modulating the tumor immune microenvironment (19).

Decrease in protective bacterial genera: The genera Serratia, Proteus and Roseomonas are reduced in abundance in patients with bladder cancer, which may suggest a potential role in tumor suppression (19).

## 3 Mechanisms of microbiome-driven pyelo-ureteral cancer

The microbiome can drive the development of UTUC through multidimensional mechanisms, which involve key biological processes such as genotoxic damage, immune-regulatory imbalance and metabolic reprogramming. The following three core dimensions are systematically analyzed:

### 3.1 Chronic inflammation and genotoxicity

#### 3.1.1 E. coli secretes colibactin to induce DNA double-strand breaks

Colibactin-producing Escherichia coli (pks+ E. coli) carrying the pks gene island can directly attack host DNA via its genotoxin colibactin. colibactin contains two cyclopropane slug structures that form alkylation adducts with DNA to form alkylating adducts that lead to DNA interstrand crosslinks (ICLs) (20). This cross-linking triggers double-strand breaks (DSBs) in DNA during its replication, activating a p53-dependent DNA damage response (DDR), and this sustained damage leads to p53 mutations and genomic instability (21). Meanwhile, some animal experiments have shown that specific adenine-colibactin adducts were detected in the colonic epithelium of mice infected with pks+ E. coli, and the level of cross-linking showed a positive correlation with tumor progression (20). In addition, colibactin can make repair defects more severe and lead to the accumulation of mutations by inhibiting the activity of the DNA repair protein FANCD2 (21).

#### 3.1.2 Alkylation damage by nitrosamine metabolites

Nitrosamines (e.g., NNK, NNAL) are metabolically activated by cytochrome P450 to generate alkyl diazene ions, which covalently bind to DNA bases (e.g., guanine) to form adducts, such as O^6-methylguanine (22). Such damage can lead to failure of mismatch repair as well as generation of point mutations in uroepithelial cells. In particular, they cause activation of oncogenes such as RAS (23). At the same time, elevated levels of nitrosamines in the urine of patients with chronic urinary tract infections and their metabolites lead to oxidative stress and mitochondrial dysfunction through preferential alkylation of mitochondrial DNA, which further contributes to the malignant transformation of the urinary tract epithelium (22).

### 3.2 Immune microenvironmental imbalance

The microbiome significantly affects the immune microenvironment of pyelo-ureteral cancer (UTUC) through metabolites, immunomodulatory molecules, and interactions with the host immune system.

#### 3.2.1 Immune checkpoint regulation and immune escape

Overexpression of B7-H4 (B7 homolog 4) is an important immunosuppressive molecule of the B7 family, which is highly expressed in UTUC, and promotes tumor immune escape by inhibiting effector T cell function. It has been found that B7-H4 enhances immunosuppressive signaling through glycolytic metabolic reprogramming in the UTUC tumor microenvironment, which in turn leads to the depletion of CD8+ T cell function (24). In addition, B7-H4 expression was significantly associated with clinical stage and poor prognosis of UTUC, also suggesting its value as a potential therapeutic target. Bidirectional regulation of the PD-1/PD-L1 axis Microbial metabolites such as short-chain fatty acids (SCFAs) and secondary bile acids can indirectly affect PD-L1 expression by regulating dendritic cell (DC) function. For example, it has been shown that Bifidobacterium bifidum reduced the expression of PD-L1 on the surface of tumor cells by lowering cholesterol levels in the tumor microenvironment, thereby enhancing the antitumor activity of CD8+ T cells (25). However, some microbial metabolites (e.g., tryptophan catabolic products) may also upregulate PD-L1 through activation of AhR receptors, creating an immunosuppressive environment (26). The urinary microbiota can modulate immune checkpoint expression via metabolites (e.g. indole-3-propionic acid, IPA).IPA up-regulates the Tcf7 gene and enhances CD8+ T-cell infiltration, which improves the efficacy of PD-1/PD-L1 inhibitors (27). In addition, tumors with high FGFR3 expression in UTUC often exhibit an ‘immune-cold’ microenvironment and are less responsive to immune checkpoint inhibitors (ICIs), whereas patients with low FGFR3 expression but abundant CD8+ T cells are more sensitive to ICIs (28), this may be related to microbiota-driven immune activation.

#### 3.2.2 Direct immunomodulation of metabolites tryptophan

Metabolism and AhR pathway activation: Gut microbes metabolize tryptophan to products such as kynurenine (kynurenine), which inhibits CD8+ T cell proliferation and promotes differentiation of regulatory T cells (Tregs) through the activation of the aryl hydrocarbon receptor (AhR).AhR signaling also induces the expression of IDO1 (indoleamine 2,3-di oxygenase 1) expression, further exacerbating tryptophan depletion and creating an immunosuppressive microenvironment (26). And in UTUC, high expression of IDO1 presents a correlation with increased Foxp3+ Treg infiltration and poor patient prognosis (29). Bile acids and NKT cell regulation: intestinal microorganisms convert primary bile acids into secondary bile acids (e.g., deoxycholic acid), which diminishes the immune surveillance function of the liver and local tumors by inhibiting the expression of CXCL16 in hepatic sinusoidal endothelial cells and reducing the recruitment of CXCR6+ natural killer T (NKT) cells (30). In contrast, abnormal bile acid metabolism may be associated with an elevated risk of tumor liver metastasis in UTUC patients. Dual role of short-chain fatty acids (SCFAs): SCFAs (e.g., butyric acid, propionic acid) regulate T-cell differentiation through inhibition of histone deacetylase (HDAC): on the one hand, it promotes Treg differentiation to maintain immune tolerance, and on the other hand, it enhances the memory function of CD8+ T cells. In UTUC, the concentration gradient of SCFAs may have determined the direction of polarization of the immune microenvironment (31).

#### 3.2.3 Dynamic balance of tumor-infiltrating immune cells regulation of T-cell subsets

On the one hand CD8+ T-cell depletion: microbial metabolites (e.g., kynurenine) can induce CD8+ T-cell depletion via the AhR signaling pathway, while upregulating the expression of inhibitory receptors such as TIM-3, LAG-3, etc (26). On the other hand reg expansion: SCFAs and AhR ligands promote Treg differentiation and inhibit anti-tumor immune response. elevated Treg ratio in tumor tissues of UTUC patients correlates with PD-1 inhibitor resistance (29). regulation of T cell subsets in turn has a dynamic impact on the tumor microenvironment. Macrophage polarization and NK cell function: microbial-derived lipopolysaccharide (LPS) induces M2-type macrophage polarization through the TLR4/NF-κB pathway, secretes IL-10 and TGF-β, and promotes tumor angiogenesis and immunosuppression. Bifidobacteria also promote NK cell infiltration into tumor tissues by increasing intestinal permeability, which in turn enhances cytotoxicity (32). However, high expression of B7-H4 in UTUC may also inhibit NK cell activation (24). Dynamics of tertiary lymphoid structures (TLS): increased CD3+ and CD8+ T cell infiltration and TLS formation in metastases correlate with response to immunotherapy. The microbiome may promote TLS formation by regulating chemokines (e.g., CXCL13), but the exact mechanism still needs to be further verified (33).

### 3.3 Metabolic reprogramming processes

The mechanisms by which the microbiome can affect renal pelvic ureteral cancer (UTUC) through metabolic reprogramming involve multi-level metabolic regulation, including direct regulation of tumor cell energy metabolism, remodeling of the tumor microenvironment through metabolites, epigenetic modifications, and host immune modulation.

#### 3.3.1 Microbiome-mediated reprogramming of metabolic pathways

Glycolysis and lactate metabolism are enhanced, and tumor cells generally suffer from the “Warburg effect”, i.e., they are preferentially supplied with energy through glycolysis rather than oxidative phosphorylation. The microbiome can exacerbate this metabolic profile through the following pathways. In terms of lactate production and utilization, certain commensal bacteria (e.g., Lactobacillus spp. in the intestinal or urinary tract) and pathogenic bacteria (e.g., Clostridium nucleatum Fusobacterium nucleatum) can produce lactate via glycolysis, which directly provides an energy substrate for tumor cells (34). In addition, microbial metabolites (e.g., butyrate) can induce the expression of lactate dehydrogenase (LDH) and α-hydroxybutyrate dehydrogenase (α-HBDH) in tumor cells, which promotes lactate production and metabolic cycling (35). Also the regulation of α-HBDH plays a role in the reprogramming of metabolic pathways. α-HBDH is one of the LDH isoenzymes, and its elevated levels are significantly associated with poor prognosis in UTUC patients (36). This enzyme may promote the adaptive metabolic process of tumor cells in a hypoxic environment by catalyzing the conversion of α-ketobutyric acid to α-hydroxybutyric acid, while releasing lactic acid to further acidify the microenvironment, which promotes invasion and immune escape (37).

#### 3.3.2 Bidirectional regulation of microbial metabolites

Microbial metabolites show a bidirectional role in tumor progression, depending on the concentration, composition of the bacterial flora and the host environment. Specifically, short-chain fatty acids (SCFAs) such as butyrate can inhibit histone deacetylases (HDACs), exerting anti-inflammatory and anti-tumor effects in many contexts. However, at high concentrations or under specific conditions, butyrate may also activate pro-inflammatory pathways such as NF-κB, potentially promoting tumor progression (38). Polyamines: polyamines (e.g., spermidine) produced by certain bacteria (e.g., Pseudomonas fragilis) may also activate the mTOR pathway and promote the tumor cell proliferation process. Hydrogen sulfide (H_2_S): H_2_S produced by sulfate-reducing bacteria can promote genomic instability by inducing DNA damage and inhibiting the mitochondrial respiratory chain (39), which can further have an effect on metabolic reprogramming.

#### 3.3.3 Clinical correlation between microbiome and α-HBDH

Some clinical studies found that elevated serum α-HBDH levels in UTUC patients were significantly correlated with tumor stage (TNM), pathological grade and lymph node metastasis (P<0.05), and were independent prognostic factors (HR = 1.36-2.04) suggesting that the α-HBDH levels may have a definite Correlation. The mechanisms may include there are microbe-host metabolic interactions: intestinal or urinary tract pathogenic bacteria (e.g., E. coli) activate the STAT3 pathway by inducing local inflammatory responses, and promote lactate accumulation and tumor cell invasion by up-regulating the expression of LDH/α-HBDH (35, 37). Metabolite feedback regulation process: high lactate environment further enriches acid-resistant flora (e.g., Lactobacillus spp.), forming a positive feedback loop that continuously drives glycolysis and α-HBDH activity, which in turn exerts a certain influence on metabolic reprogramming process, tumor staging (TNM), pathological grading and lymph node metastasis.

## 4 Application of microbiome in the diagnosis and treatment of UTUC

### 4.1 Diagnostic and prognostic markers

Upper urinary tract uroepithelial carcinoma (UTUC) is a highly invasive tumor, which is difficult to diagnose at an early stage, has a high rate of recurrence and a poor prognosis, and the existing non-invasive diagnostic methods (e.g., urinary cytology, etc.) have insufficient sensitivity.

In recent years, human microbiome studies have provided new directions for the diagnosis and treatment of UTUC, including the combination of urinary microbial markers (e.g., E. coli-specific gene clusters) with circulating tumor DNA (ctDNA) assays to enhance diagnostic sensitivity, and the association of indices of intestinal bacterial diversity (e.g., Simpson’s index) with the response rate to chemotherapy and the risk of recurrence. Urinary microbial markers are of value in UTUC diagnosis. potential value of E. coli-specific gene clusters Gene clusters with respect to biological function. For example, the f9 flagellar gene cluster of E. coli (e.g., f9A, f9G) promotes biofilm formation at low temperatures (20 °C) and its adhesion proteins are specific for Galb1-3GlcNAc glycosylation, which may be involved in UTUC by adhering to urinary epithelial cells and thus in UTUC (40). In addition, its S bacterial mycelium (sfa) and F1C bacterial mycelium (foc) gene clusters are highly conserved in pathogenic E. coli, which may have an impact on UTUC progression by mediating bacterial colonization and immune escape (41).

The diagnostic aspect of the disease is that the expression level of E. coli-specific gene clusters in urine can be tested to identify pathogenic bacterial infections associated with UTUC, thus aiding in the diagnosis. For example, the detection of the f9 gene cluster can be combined with urine DNA sequencing technology, which can improve the sensitivity to UTUC-associated infections (40). Meanwhile breakthroughs in ctDNA detection technology have also been of value in the diagnostic process of UTUC. For example, gene mutation and methylation markers. tert promoter mutations (e.g., C228T, C250T) and FGFR3 mutations (e.g., S249C) were detected in urine ctDNA of UTUC patients at a rate of 39.3% and 16.1%, respectively, and then combined with urinary cytology testing can increase the sensitivity to 78.6% and the specificity to 96% (42). Therefore, the combined application of urinary microbial markers (e.g., E. coli gene cluster) and ctDNA can capture tumor and microbiome information in multiple dimensions, which can provide certain value for the diagnosis and prognosis of disease regression.

The human microbiome is also valuable in UTUC prognosis, and can be used as a prognostic marker to assess the prognosis and efficacy of the disease. In terms of gut flora diversity index (Simpson index) and chemotherapy response rate. High intestinal flora diversity (high Simpson’s index) was positively correlated with chemotherapy response rate. For example, increased abundance of specific flora (e.g., Eubacterium limosum) in highly diverse patients may enhance the efficacy of chemotherapeutic agents such as cyclophosphamide by modulating the immune response (e.g., enhancing TH17 cell activity). The mechanism of action is that intestinal flora can regulate the tumor microenvironment through metabolites (e.g., short-chain fatty acids) and enhance drug permeability; at the same time, certain strains (e.g., Bifidobacterium limosum) can promote the efficacy of immune checkpoint inhibitors by activating dendritic cells and CD8+ T cells (43). Meanwhile, some scholars have shown in their studies that low bacterial diversity (low Simpson’s index) may present a correlation with the risk of UTUC recurrence. Patients with low Simpson’s index (low flora diversity) had a significantly higher risk of postoperative recurrence. Meanwhile, patients with postoperative recurrence of UTUC have increased abundance of pathogenic bacteria (e.g., Escherichia-Shigella) and decreased probiotic bacteria (e.g., Bifidobacterium) in their intestinal flora (44), and one study in patients with septic shock showed that a low Shannon’s diversity index (<3.0) was correlated with an elevated mortality rate (OR = 2.04). Suggesting that flora imbalance may promote tumor recurrence through chronic inflammation (45), whereas in UTUC, the 5-year survival rate of patients with low diversity was significantly lower than that of patients with high diversity (44.07% vs. 81.82%) (46), suggesting that a low Simpson’s index may present a correlation with the risk of recurrence in UTUC.

### 4.2 Therapeutic strategies

For antibiotic-targeted therapy, colibactin-producing E. coli (pks+ E. coli) induces DNA damage through its genotoxin colibactin, which forms DNA interstrand cross-links (ICLs) and double-strand breaks (DSBs) (21), and such damage can trigger the homologous recombination repair (HR) mechanism, leading to resistance of tumor cells to platinum-based drugs such as oxaliplatin. Platinum drugs themselves exert cytotoxicity through the formation of DNA adducts, and enhanced HR repair may diminish their efficacy (47). In addition, colibactin-induced oxidative stress and abnormalities in lipid metabolism may further promote immunosuppression and chemoresistance in the tumor microenvironment (48). For example, in colorectal cancer models, removal of colibactin-producing E. coli reduces the activation of DNA damage repair-related genes (e.g., ATM, FANCD2), thereby restoring sensitivity to platinum-based drugs (48). In UTUC, there is still a need to develop selective antibiotic or phage therapies against pks+ E. coli to avoid the destruction of commensal flora by broad-spectrum antibiotics. Simultaneous development of therapeutic strategies for resistance monitoring is also needed, as long-term use of antibiotics may accelerate the evolution of resistant strains, and dynamic monitoring of the combined microbiome is required. In terms of probiotic intervention, the immunomodulatory role of Lactobacillus reuteri in restoring the Th1/Th2 balance should not be overlooked.L. reuteri promotes the secretion of Th1-type cytokines (IFN-γ, IL-12) and inhibits the secretion of Th2-type cytokines (IL-4, IL-12) through the secretion of metabolites (e.g., short-chain fatty acids, vitamin B12) and the regulation of the function of dendritic cells. cytokines (IL-4, IL-10), thus reversing the Th2 bias in the tumor microenvironment (49) and playing a regulatory role in immune homeostasis. It also secretes antimicrobial substances, and its production of reuterin (a broad-spectrum antimicrobial agent) can inhibit pathogenic bacterial colonization, which can have a remodeling effect on the structure of the flora (50). Some scholars have found in animal models that supplementation with L. reuteri can significantly increase CD8+ T cell infiltration and IFN-γ levels, thereby inhibiting tumor growth (51). However, there are still some challenges in probiotic intervention therapy, with specificity differences between different strains of probiotics, and differences in the immunomodulatory effects of different L. reuteri strains, which need to be screened for highly active strains to further improve therapeutic efficacy (52). However, the gold standard for diagnosing UTUC remains professional pathological diagnosis during and after surgery. Antibiotic intervention can lead to pathogen resistance and disruption of the body’s own microbial flora. Therefore, it is essential to acknowledge that antibiotic intervention and other such measures can affect the body’s microbial flora. Consequently, a comprehensive and holistic evaluation of the use of antibiotics in relation to the human microbial flora should be maintained.

## 5 Current challenges and future directions

Upper urinary tract samples are difficult to obtain and microbial abundance in UTUC samples is extremely low, and second-generation sequencing (NGS) is susceptible to contamination, which affects the analysis of results. Meanwhile, the existing studies are mostly confined to the analysis of bacterial flora composition and lack the integration between metabolomics and transcriptomics. Moreover, the causal mechanism is still unknown, and whether the flora change is a driver or a concomitant phenomenon of cancer still needs to be further verified (53, 54). Meanwhile, there are certain research limitations for UTUC, firstly, the low incidence of UTUC makes it more difficult to carry out large-scale cohort studies, and secondly, there is also great heterogeneity interference, and UTUC caused by different etiological factors (e.g., smoking, etc.) may be accompanied by different inter-flora characteristics (55). However, it can be found by liquid chromatography-mass spectrometry (LC-MS) analysis that metabolites (e.g., tryptophan derivatives) in the urine of patients with UTUC differ significantly from those of the healthy population and patients with bladder cancer, which makes UTUC different from other epithelial cancers of the urinary tract (56). Currently, there is a lack of large-scale trials validating microbiome-based biomarkers or engineered probiotics in urothelial carcinoma, so this challenge still needs to be validated and confirmed in future research.

In terms of clinical translation, the development of diagnostic markers for UTUC is simultaneously valuable. In terms of gene mutation and methylation, TERT promoter mutation, FGFR3 mutation and NRN1 gene methylation in urinary DNA were confirmed to be potential markers for UTUC, with a combined detection sensitivity of 94% and specificity of 93% (57). Meanwhile miRNAs such as miR-664a-3p and miR-423-5p in serum can distinguish UTUC patients from healthy controls with AUC values exceeding 0.8 (58), which can be considered to have good diagnostic ability.

In the future, cross-ethnic multicenter studies can be promoted to include different populations such as Asia, Europe and the United States, and stratified analyses can be performed by combining diet, antibiotic and other use, and genetic backgrounds (e.g., Lynch syndrome), in order to clarify the ethnic specificity of the microbiome (59). Multi-omics integration models can also be further developed to combine microbiome, metabolome and genomic data to construct early diagnosis and prognosis prediction models for UTUC. For example, the random forest model based on intestinal flora in pancreatic cancer study had an AUC of 0.97, suggesting the feasibility of a similar strategy in UTUC. At the same time, the construction of animal and *in vitro* models for UTUC has become important in the study of UTUC. Meanwhile, indole derivatives (e.g., indolephenol sulfate) produced by intestinal flora metabolism promote inflammatory responses and oxidative stress in the renal pelvic microenvironment through the activation of the aromatic hydrocarbon receptor (AhR), which has a certain impact on the progression of UTUC (60). further in-depth explorations of the bidirectional intestinal-renal axis regulation are needed to study how intestinal flora metabolites affect the renal pelvic immune microenvironment through the circulatory system, and to screen the key regulatory nodes as therapeutic targets. Bifunctional probiotics can also be developed, such as expressing both anticancer molecules (e.g., immune checkpoint inhibitors) and enzymes degrading urotoxins, or precisely editing the virulence genes of oncogenic bacteria using CRISPR technology (61). To better validate the microbiota-driven mechanisms proposed in UTUC, the establishment of disease-specific experimental systems is essential. Recent progress in 3D-bioprinted urological cancer models has offered new opportunities for more physiologically relevant studies of tumor–microbiome interactions (62). Moreover, advances in immunotherapy, particularly adoptive cell transfer strategies, provide mechanistic insights into checkpoint blockade pathways such as PD-1/PD-L1 and B7-H4, which are heavily discussed in the context of microbiota-modulated immune suppression (63). Similarly, there is a lack of longitudinal and multi-omics studies; it is difficult to determine the causal relationship of microbiome changes; and there is a lack of UTUC-specific mitochondrial or organoid models, which should be focused on and addressed in microbiome and clinical disease research.

## 6 Summary

The pathogenesis of UTUC is closely linked to the microbiome, and the mechanisms involve a variety of biological processes. Pathogenic bacteria-mediated genotoxicity: colibactin-producing E. coli leads to genomic instability by inducing DNA double-strand breaks and alkylation damage; nitramine metabolites promote malignant transformation through mitochondrial dysfunction. Imbalance in the immune microenvironment: microbial metabolites (e.g., kynurenine, secondary bile acids) inhibit CD8+ T cell function and promote Treg differentiation through the AhR pathway and immune checkpoint regulation, creating an immunosuppressive microenvironment; B7-H4 overexpression further exacerbates immune escape. In terms of metabolic reprogramming: the microbiome can drive metabolic adaptation of tumor cells by enhancing glycolysis, lactate accumulation, and α-HBDH activity, and correlates with clinical staging and poor prognosis. In terms of diagnosis and treatment, the combined detection of urinary microbial markers and ctDNA can enhance the sensitivity and specificity of early diagnosis of UTUC; the diversity index of intestinal flora and the abundance of specific genera (e.g., Bifidobacterium) provide a new direction for the assessment of prognosis of UTUC and the efficacy of chemotherapy. However, the current study faces challenges such as insufficient sample size, unknown causal mechanisms and multiple technical limitations. In the future, in-depth mechanism exploration through multi-center cohort studies, multi-omics integration (e.g., microbiome-metabolome-genome), bidirectional regulatory relationship of the gut-kidney axis, and targeted intervention strategies (e.g., probiotic therapies, CRISPR editing of causative organisms) are needed to further promote clinical translation. The study of human microbiome has opened up a new way of thinking for precision diagnosis and treatment of UTUC, but its clinical application still needs to be further verified and optimized.
